# Ethnic differences in the prevalence of online behaviors in adolescents in the southern regions of siberia

**DOI:** 10.1192/j.eurpsy.2021.1498

**Published:** 2021-08-13

**Authors:** N. Semenova, L. Evert L, Y. Kostyuchenko, S. Tereshchenko

**Affiliations:** 1 Scientific Research Institute For Medical Problems Of The North, Federal Budgetary Scientific Institution «Federal Research Centre «Krasnoyarsk Scientific Centre of Siberian Division of Russian Academy of Sciences», Krasnoyarsk, Russian Federation; 2 Research Institute For Medical Problems In The North, Federal Research Center “Krasnoyarsk Scientific Center of the Siberian Branch of the RAS”, Krasnoyarsk, Russian Federation

**Keywords:** Internet, prevalence, ethnic, adolescents

## Abstract

**Introduction:**

An urgent problem all over the world is the growing number of adolescents with maladaptive (Internet addicted) Internet use.

**Objectives:**

To study the prevalence of various types of online behavior in adolescents in the southern regions of Siberia (Caucasians and Mongoloids).

**Methods:**

4351 adolescents aged 12-18 in the city of Krasnoyarsk and the city of Abakan (Republic of Khakassia) were surveyed. Ethnicity is determined by the nationality of the mother. Online behavior was studied using the Chen Internet Addiction Scale (CIAS): adaptive internet use (API) – 27-42 points, non-adaptive (NPI) – 43-64 points and pathological (PPI) ≥ 65 points. The indicators were compared in 2 groups: Caucasians and Mongoloids. The program “Statistica 12” was used, the percentage of the share, the significance of the differences (p) and the values of the Pearson χ^2^ test were indicated.

**Results:**

Caucasians by their mothers accounted for 3663 (84.2%) and the share of Mongoloids reached 688 (15.8%). AIP was recorded in 44.0% of Caucasians and 7.9% of Mongoloids (p <0.0001; χ2 = 1474.99), NPI was recorded in 34.7% of Caucasians and 6.2% of Mongoloids (p <0.0001; χ2 = 1084.65), PPI was found in 5.5% of Caucasians and 1.7% of Mongoloids (p <0.0001; χ2 = 90.49).

**Conclusions:**

Ethnic features of the prevalence of online behavior in adolescents in the southern regions of Siberia include a higher frequency of NPI and PPI in Caucasians compared to Mongoloids. The reported study was funded by RFBR according to the research project № 18-29-22032\18.

**Conflict of interest:**

The reported study was funded by RFBR according to the research project № 18-29-22032\18.

